# Single-Crystal MoO_3_ Micrometer and Millimeter Belts Prepared from Discarded Molybdenum Disilicide Heating Elements

**DOI:** 10.1038/s41598-018-34849-y

**Published:** 2018-11-13

**Authors:** Xiaolong Hou, Juntong Huang, Mingqiang Liu, Xibao Li, Zhihui Hu, Zhijun Feng, Meng Zhang, Junming Luo

**Affiliations:** 1 School of Materials Science and Engineering, Nanchang Hangkong University, Nanchang, Jiangxi Province 330063 P. R. China; 2 The State Key Laboratory of Refractories and Metallurgy, Wuhan University of Science and Technology, Wuhan, 430081 China

## Abstract

Single-crystal MoO_3_ micrometer to millimeter even centimeter belts were prepared via a novel route of oxidizing a discarded molybdenum disilicide heating element at 1000 °C for 3 h. The morphology and structure features, and growth mechanism of the products were evidently investigated by X-ray diffraction, Fourier transform infrared spectroscopy, X-ray photoelectron spectroscopy, scanning electron microscopy, and transmission electron microscopy. The results indicated that the powdery and fibrous products were typical α-MoO_3_ belt-like structures which size could develop from micrometer to several millimeter even centimeter in length and up to 0.5 mm in width. It should be formed preferentially along the [001] direction via layer by layer growth to form 1-D single MoO_3_ belts by vapor-solid mechanism. Thermal and luminescence properties of the products were revealed by thermogravimetric analysis and differential thermal analysis and photoluminescence spectra that the resultant α-MoO_3_ belts had good thermal stability and characteristics of luminescence with a central peak at 481 nm. The MoO_3_ belts are of good potential being applied to luminescent and high temperature devices.

## Introduction

Molybdenum trioxide (MoO_3_) usually exhibits three polymorphic structures, orthorhombic (α-MoO_3_), monoclinic (β-MoO_3_), and hexagonal phase (h-MoO_3_), in which the former is the only thermodynamically stable phase^1,2^. α-MoO_3_ has attracted intense interest due to its distinctive layer structure, formed by bilayer sheets of MoO_6_ octahedra stacked together *via* van der Waals forces^2–4^. Because of its intrinsic structural anisotropy and the change ability in the oxidation state of molybdenum ion, α-MoO_3_ is considered to have many promising applications in lithium-ion batteries^5–7^, gas sensors^8,9^, catalysis^10,11^, field emission^12^, photochromic and electrochromic devices^13,14^.

α-MoO_3_ phase is known to develop into one-dimension (1D) structures (wires, tubes, belts)^15^. The past few years have witnessed significant advances in the synthesis of belt-like α-MoO_3_ using various methods, such as solution method^16–18^, hydrothermal method, thermal evaporation, flame synthesis using Mo wire mesh as the source as well as CH_4_, H_2_, and air as the supply fuels^2^. However, most of resultant products are nanometers or micrometers in size. The preparation of single-crystal belts on millimeter to centimeter-scale (or macroscopic) has been seldom reported^19^.

Here we present a novel route to prepare single-crystal α-MoO_3_ micrometer to millimeter even centimeter belts *via* oxidizing a discarded molybdenum disilicide heating element on the top of corundum crucible at 1000 °C for 3 h. The belt-like products are formed in the bottom of corundum crucible separated away from the raw materials, making it easy to collect. The resultant belts were fully characterized. Furthermore, their thermal and photoluminescence properties were tested. Based on the results, the relevant growth mechanisms of the belts were discussed, and the blue-indigo optical emissions of the α-MoO_3_ belts were revealed. In this way, we could recycle the malfunctioned MoSi_2_ heating elements for the production of MoO_3_ single crystal belts for the design and application of blue-indigo optoelectronic devices.

## Results and Discussion

### Phases, chemical compositions and states of the resultant products

**Figure 1:**
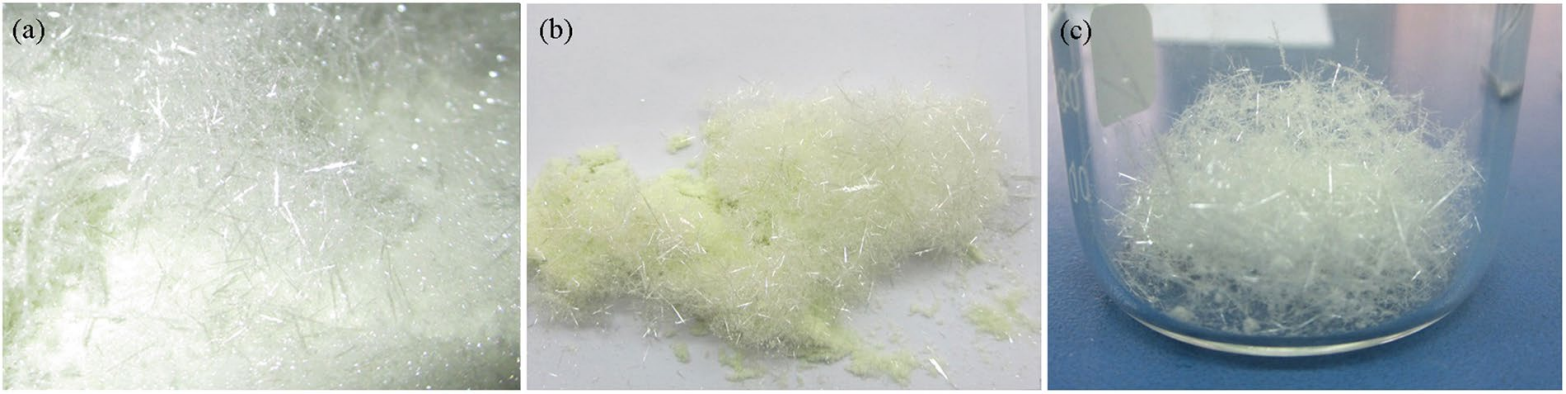
Optical Photograph of the samples: belts-like on the top, powder in the bottom.

A light green products with two parts of powdery and fibrous crystals were visually seen in the bottom of the corundum crucible after 3 h of reaction at 1000 °C in air (Fig. 1a), and the powders were covered by the fiber-like products. It is obviously that the fibrous products were several millimeters even up to centimeter long (Fig. 1c). Both powdery and fibrous products were collected respectively and subjected to the characterizations using XRD, FTIR, XPS, SEM and TEM.

**Figure 2:**
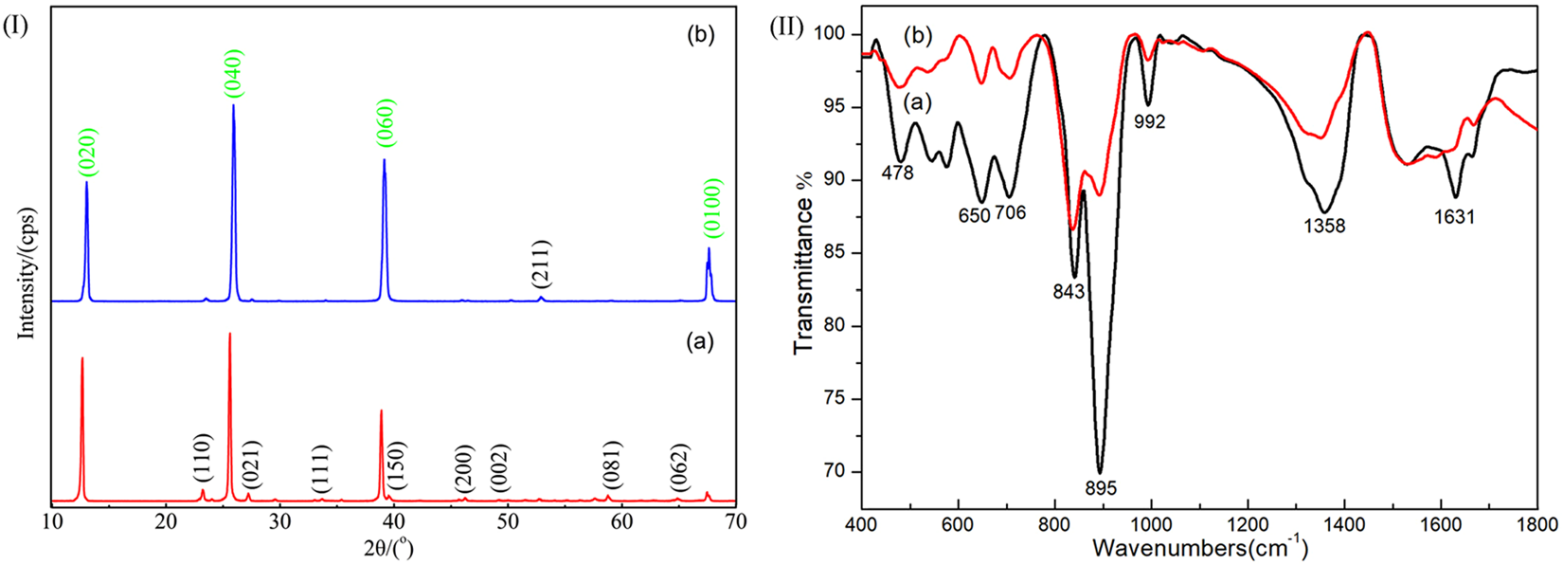
(I) X-ray diffraction pattern of products: (**a**) powders and (**b**) fibers; (II) FT-IR spectra of the powders (**a**) and fiber (**b**) sample.

The XRD results (Fig. 2I) indicated that the powdery and fibrous products only had the thermodynamically stable phase α-MoO_3_ (JCPDS card No. 05–0508) without impurity peaks, suggesting the high purity phase of the resultant α-MoO_3_. The obvious intensities of the (0k0) diffraction peaks with k = 2, 4 and 6 revealed anorientation along the [010] direction ascribed to its packed double layered crystal structure^9^. Furthermore, the intensities of other diffraction peaks of MoO_3_ in powdery product were much higher than those in fibrous one.

Their FT-IR spectra are shown in the Fig. 2II, which were obtained the range of wave numbers from 400 to 1800 cm^−1^. The characteristic peaks at 478, 650, 895 and 992 cm^−1^ were arisen from the fundamental vibrational modes of Mo=O^20^. The peak at 843 cm^−1^ was due to the characteristic vibration of Mo-O-Mo bridging bonds. The peak at approximately 1358 cm^−1^ was assigned to the vibration mode of the Mo–OH bond whereas the peak at 1631 cm^−1^ was associated with the δ (O-H) vibration of adsorbed water^17^. As for the absorption peaks between 478 and 650 cm^−1^ were because of the stretching vibration of the O atom link to three Mo atoms^17,18,21^.

**Figure 3:**
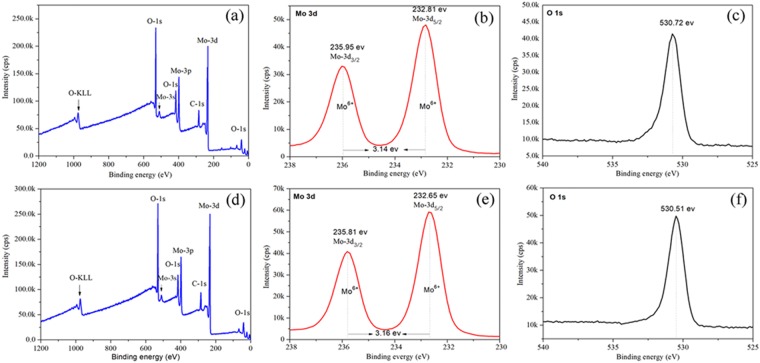
The X-ray photoelectron spectra (XPS) of the survey scan results, Mo-3d and O-1s in the powdery products (**a**–**c**) and fibrous products (**e**,**d** and **f**).

Photoelectron spectroscopy (XPS) was further employed to determine the chemical compositions and chemical states of products. Figure 3a,d show the survey scan X-ray photoelectron spectrum of the two parts products respectively, revealing the peaks assigned to molybdenum (Mo), oxygen (O), a trace amount of carbon (C) and O-KLL Auger peaks. The peak at around 284.8 eV was corresponded to C-1s fine structure singlet of carbon, which was used as a charge correction reference in this XPS test. The high resolution narrow scan XPS spectra of Mo-3d and O–1 s core levels were further recorded in order to identify the chemical states of elements. The narrow scan XPS spectrum of Mo-3d core level (Fig. 3b) in powdery products showed the existence of two well resolved spectral lines. This was ascribed to the spin-orbit split of Mo-3d levels with the binding energy at 232.81 eV and 235.95 eV relate to Mo-3d_5/2_ and Mo-3d_3/2_, respectively. The energy gap between Mo-3d_5/2_ and Mo-3d_3/2_ is 3.14 eV, and their corresponding integral area ratio was about 3:2, supporting the presence of single + 6 oxidation state in molybdenum^22^. The peak at 530.72 eV (Fig. 3c) was assigned to the O^2-^ ions in the MoO_3_. Thus, these binding energy peak positions and energy gap were in highly consistent with the past reports for MoO_3_^16,22,23^. Meanwhile, the fibrous products exhibited nearly the same results to the powdery one. So it is believed that the powdery and fibrous products were MoO_3_.

### Morphology and microstructure of the resultant products

**Figure 4:**
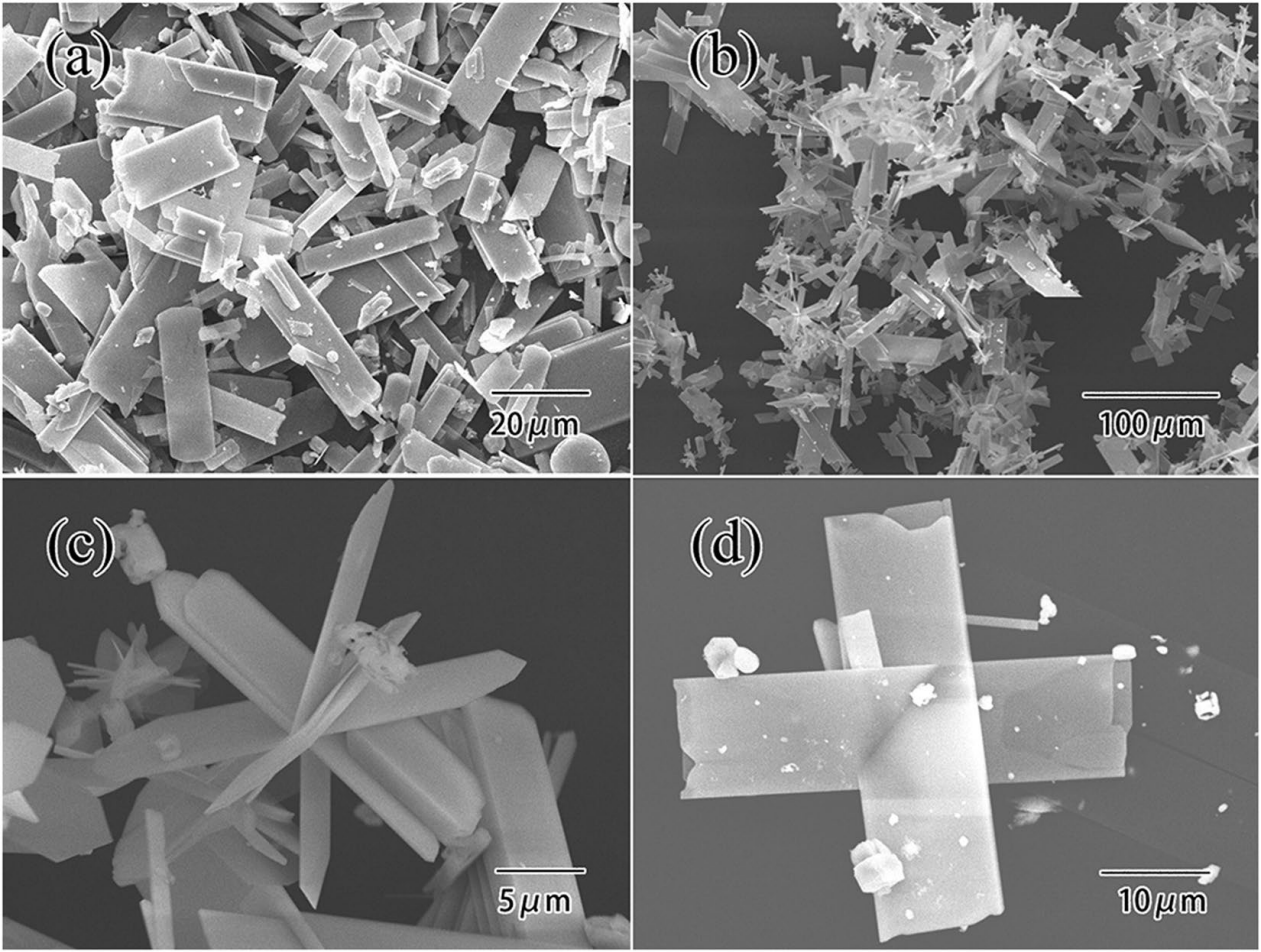
SEM images of powdery products.

The morphologies of the two parts products can be observed clearly under SEM. Figure 4 shows the different magnifications SEM micrographs of powdery MoO_3_ having different shape, length and width. Figure 4a displays a great number of lamelliform MoO_3_ crystals, which had clear outline and smooth surface, length was range from 10 μm to 50 μm. Figure 4b displays that the product was composed of gathered lamelliform MoO_3_ microbelts with various sizes, which should be caused by the inhomogeneous nucleation of single crystalline MoO_3_ particles^17^. Figure 4c,d show interspersed growth of MoO_3_ to form flower-like and cross-shaped structures with the size of 15~40 μm long, and 5~12 μm wide. The surface of the MoO_3_ microbelts was smooth.

**Figure 5:**
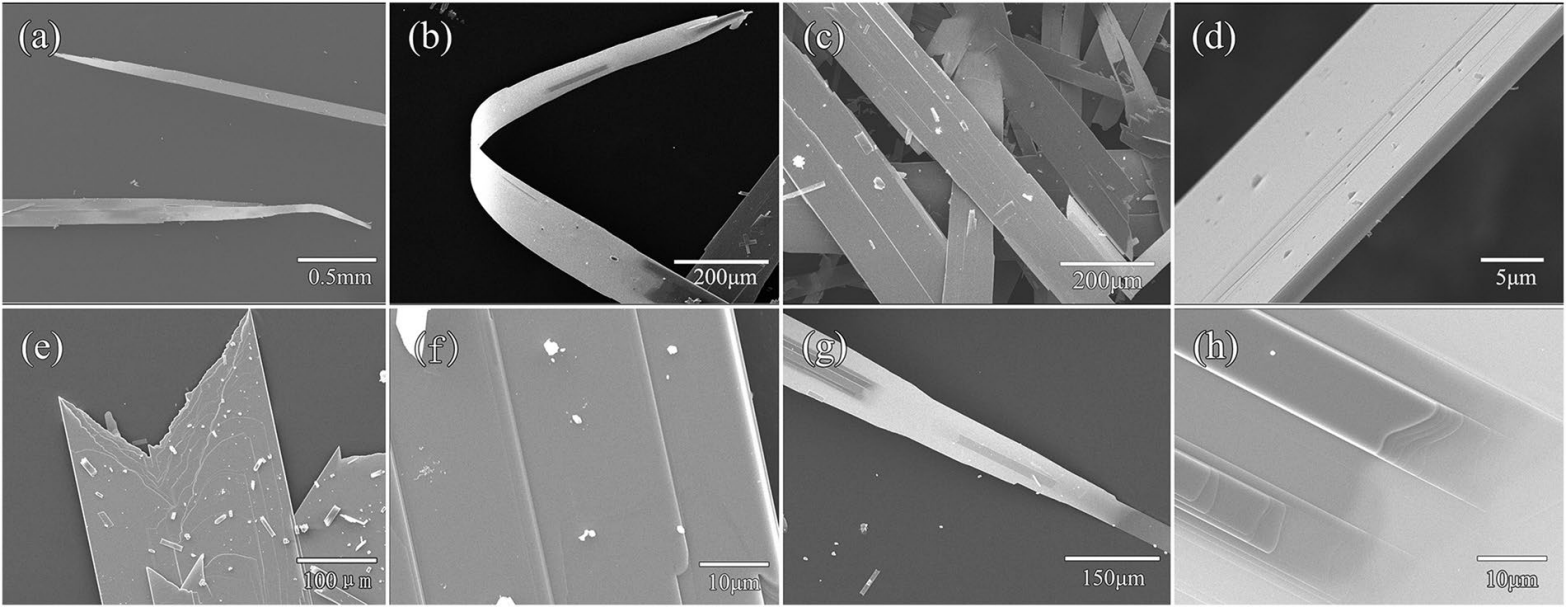
SEM images of belt-like sample at different magnification rates.

The SEM images of fibrous MoO_3_ products are shown in the Fig. 5, indicating their belt-like structures. Figure 5a–c shows that the large size MoO_3_ belts were 0.2 mm wide, and up to several centimeters long. This should be the largest size MoO_3_ belts reported. The MoO_3_ belt was highly curved (Fig. 5b), indicating good flexibility. The MoO_3_ belts decreased gradually in size and had a needle-like tip at its terminal (Fig. 5a,b). After further enlarging the image, the MoO_3_ belts had a smooth surface and a neat edge with the thickness of about 2 μm (Fig. 5d). Sometimes, the belts with a zigzag edge composed of triangles having tip angles were found (Fig. 5e), and their surfaces had a layered structure in both horizontal and vertical directions (Fig. 5e–h). Further enlargement of Fig. 5e, it shows that the MoO_3_ belt grew layer by layer and started horizontally in the middle of the belt. The thickness of each layer was approximately tens of nanometers, and the horizontal spacing of each layer was approximately 10–15 μm. Observed from Fig. 5g, the surface of the MoO_3_ belt had a distinct step shape. Enlarging the image (Fig. 5h), it could be observed that the surface of the MoO_3_ belt had two row layered growth structures with several vertical layers on each row.

**Figure 6:**
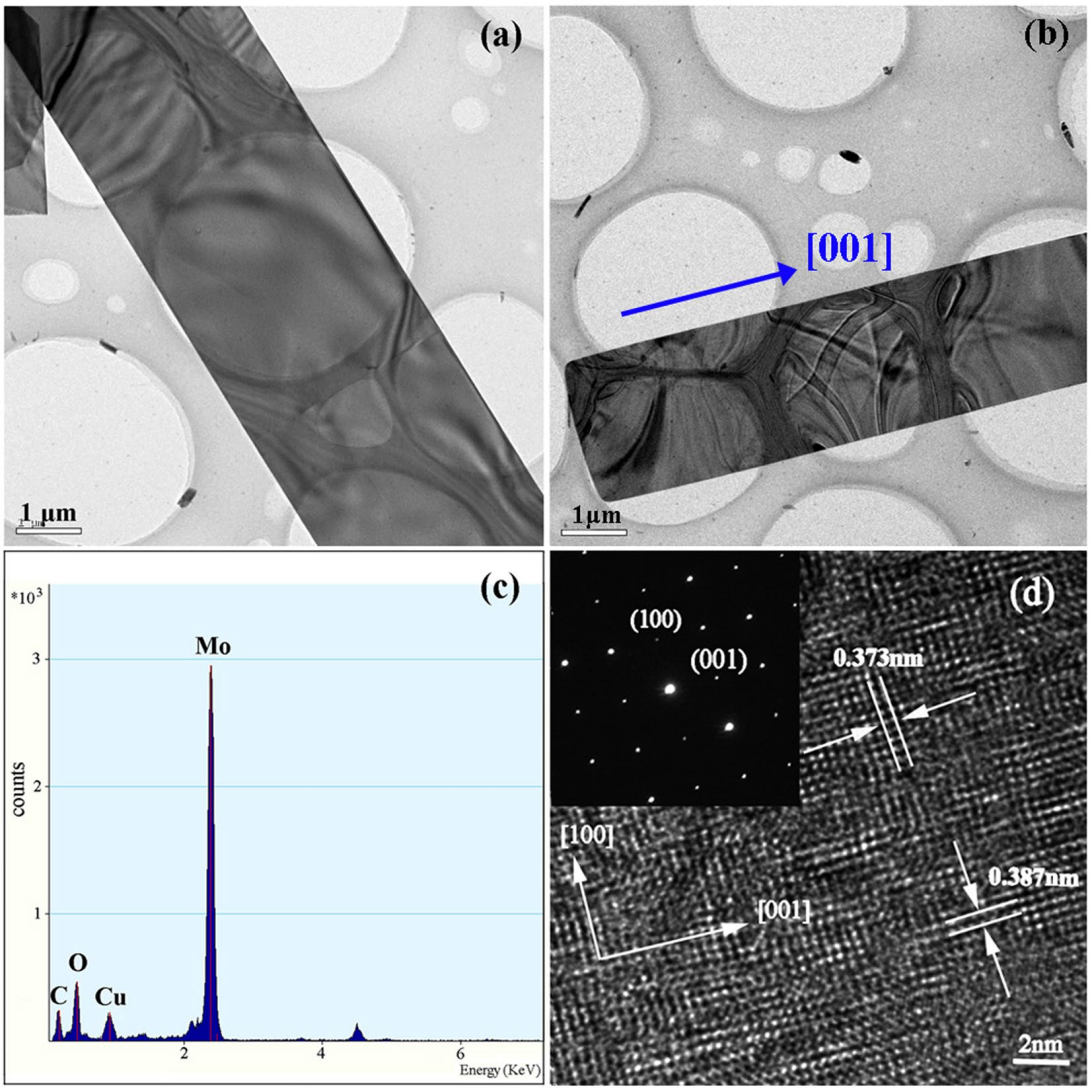
(**a**,**b**) TEM images of single microbelt, (**c**) EDS pattern of single microbelt, (**d**) HRTEM image and the inserted SAED pattern of microbelt.

The detailed crystalline structure and growth direction of MoO_3_ belt-like products were further detected by TEM and high resolution TEM (HRTEM). Figure 6a,b showed the product with a typical belt-like geometry as well as a uniform width along its length. EDS was also recorded to determine the chemical composition of α-MoO_3_ belts (Fig. 6c). Except the C and Cu peaks from TEM grid, just the elements of Mo and O were detected in the belts. Thus, both XRD and EDS analyses revealed that the pure α-MoO_3_ product had been successfully synthesized. The HRTEM lattice images (Fig. 6d) revealed the microbelts possessed a well-defined crystal structure, and the spacing between the two sets of parallel fringes was 0.373 nm and 0.387 nm, corresponding to the two vertical planes (001) and (100) of α-MoO_3_^14,24–26^. Therefore, the HRTEM image and SAED pattern (Fig. 6d), recorded with the incident electron beam perpendicular to the nanobelt face, confirmed that the microbelts were composed of crystalline orthorhombic α-MoO_3_ with top/bottom surfaces of (010) and a growth direction of [001], similar to previously reported^2,9,26^.

### Growth mechanism

**Figure 7:**
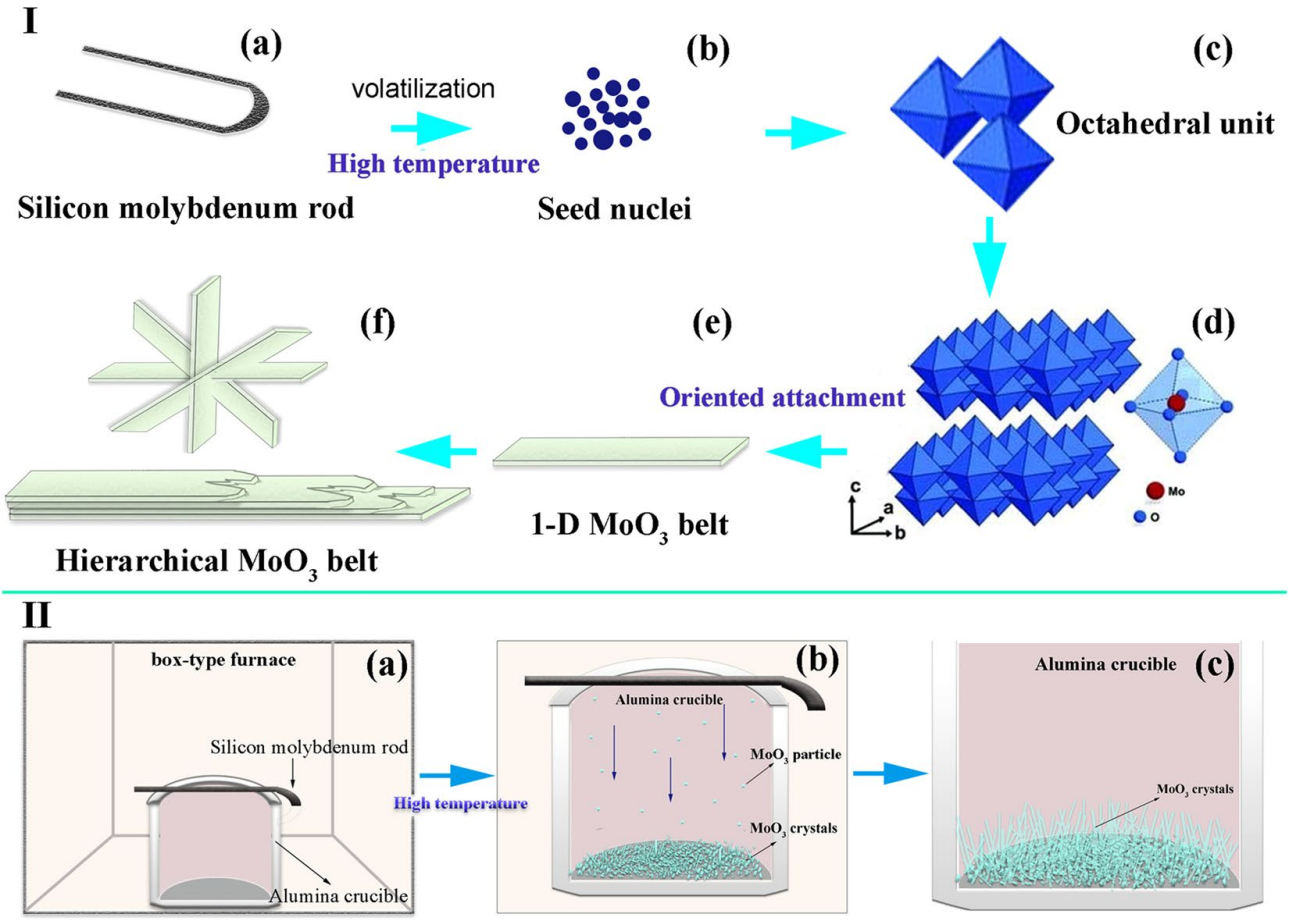
The (I) microscopic and (II) macroscopic growth mechanism of single MoO_3_ crystal.

Based on the above observations and analysis, the formation mechanism and growth process of α-MoO_3_ belts prepared via the route of oxidizing a discarded molybdenum disilicide heating element at 1000 °C can be schematically illustrated in Fig. 7 and described as follows. At 1000 °C, molybdenum disilicide heating element should be oxidized to form MoO_3_ and SiO_2_, according to eqn (1). It is known that the melting point and boiling point of MoO_3_ are 795 °C and 1155 °C, lower than those of SiO_2_, 1650 °C and 2230 °C, respectively. Therefore, in this work, MoO_3_ must be volatilized, condensed and deposited in the bottle of corundum crucible whereas SiO_2_ should be formed on the surface of heating element.

1 $$2MoS{i}_{2}+7{O}_{2}\to 2Mo{O}_{3}+4Si{O}_{2}$$

Firstly, at the test temperature, significant MoO_3_ vapor would be generated in a mass, and after a cooling process the MoO_3_ vapor would be condensed and the MoO_3_ seed nuclei generated with the octahedral structure (Fig. 7Ia-c), followed by the formation of a proto-belt of MoO_3_ with the vapor-solid mechanism. To form these proto-belts, the initial growth in the width direction should be quicker than along the thickness direction. Because the MoO_3_ crystal have an orthorhombic system with the lattice parameter: a = 3.96 Å, b = 13.86 Å and c = 3.70 Å, its growth along c direction would be favored because of a lowest surface energy of the (001) plane and the formed belt-like structure should be resulted from large differences in the close-packing rate among the (100) and (010) planes. The α-MoO_3_ has an intrinsic tendency to grow an anisotropic structure because the planar growth rate along axis of the crystal has the following sequence {001} > {100} > {010}^27,28^. Therefore, after the formation of the proto-belt of MoO_3_, it should be grown preferentially along the [001] direction to form 1-D single MoO_3_ belts (Fig. 7Id-e). As the reaction progresses, the MoO_3_ microbelts were formed by layer by layer growth, eventually forming ribbon-shaped, cross-shaped, and flower-like MoO_3_ structures (Fig. 7If). The macroscopic growth mechanism of single MoO_3_ crystals in this study were as shown in Fig. 7-II(a-c). In the VS mechanism, the size of MoO_3_ crystal was proportional to growth time and growth rate which depended on evaporation rate and system pressure^29^. So the molybdenum disilicide rod was oxidized and sublimated to form MoO_3_ gas phase molecules at high temperature, which were then deposited on the bottom of the crucible and grown to form MoO_3_ crystals. The initially formed seed crystals had enough time and rapid growth rate to develop MoO_3_ belts up to millimetres or even centimeters, while the seed crystals generated at the later period at cooling down with short time and slow growth rate just were grown forming small microbelts.

### Thermal and photoluminescence properties

**Figure 8:**
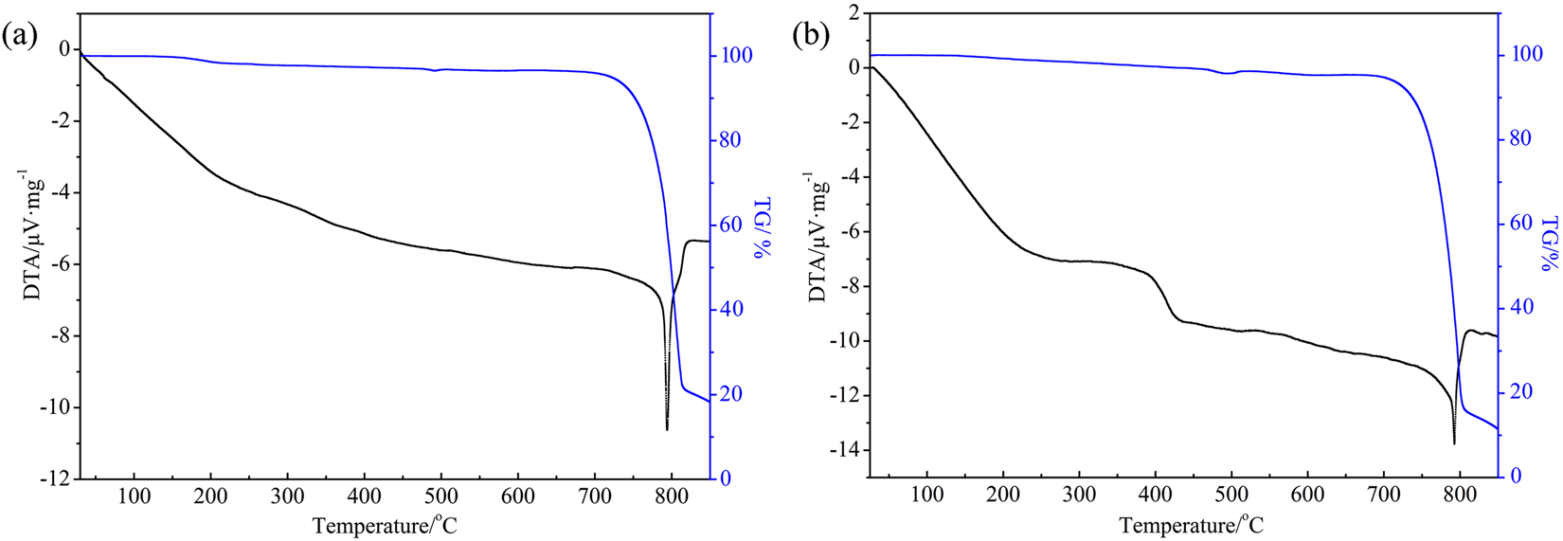
TG-DTA curve of (**a**) the powdery microbelts and (**b**) fibrous α-MoO_3_ millimeter-belts.

The powdery and fibrous products were confirmed to be α-MoO_3_ microbelts and millimeter-belts. In order to understand their thermal stability and relative weight loss, thermogravimetric and differential thermal analysis (TG-DTA) were performed in air atmosphere. Figure 8a shows the TG-DTA curves of powdery α-MoO_3_ microbelts, initial weight was around 3.293 mg, and its weight had hardly significant change before 700 °C as well as the TG curve without ant stage decomposition, and only a loss of weight was about 4.03% due to the evaporation of water molecules in the product, so that powdery α-MoO_3_ microbelts had good thermal stability before 700 °C. Whereas, there was an intense endothermic peak at about 795 °C in the DTA curve which was corresponding to the temperature of melting point of α-MoO_3_. At this temperature, there was a dramatic weight loss of about 57.27% in the TG curve, resulted from the sublimation of MoO_3_^21,28^. Similarly, fibrous products with α-MoO_3_ millimeter-belts had similar results (Fig. 8b), but it has two inapparent endothermic peak at about 237 °C and 383 °C in the DTA curve, and a loss of weight about 5.17% should be due to the evaporation of water molecules in the product, and a loss of weight about 66.3% due to sublimation of MoO_3_ at 795 °C. It is verified that the two kinds of belts had favourable flame retardancy and thermal stability before 700 °C.

**Figure 9:**
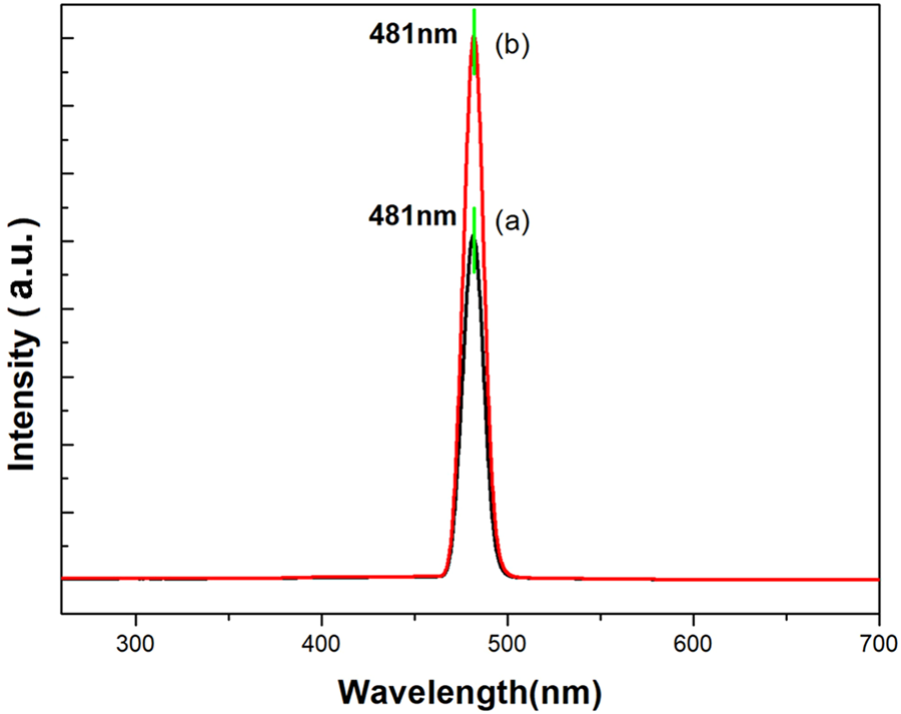
Photoluminescence spectra of (**a**) the powdery microbelts and (**b**) fibrous α-MoO_3_ millimeter-belts.

Figure 9 shows the representative photoluminescence (PL) spectra of the two kinds of α-MoO_3_ belts in the wavelength range from 250 to 700 nm under the excitation of 240 nm at room temperature. Both of the powdery microbelts and millimeter-belts exhibited a strong emission from around 465 nm to 495 nm with a central peak at 481 nm (~2.578 eV) located in the blue-indigo spectral range, corresponding to the recombination between electron and hole of the α-MoO_3_ belts. But the fibrous α-MoO_3_ millimeter-belts had stronger peaks than the MoO_3_ powder, which should be due to the difference between the crystal size and the degree of edge distortion. It could be valuable for the future potential applications in optoelectronic devices^29^.

In conclusion, α-MoO_3_ belt-like structures were obtained by oxidizing a waste molybdenum disilicide heating element at 1000 °C. The oxidized products were consisted of two parts, powdery and fibrous crystals, in which the sizes of the former were 15~40 μm long, some of them could grow into flower-like and cross-shaped structures, and the later could develop to millimeter even centimeter long. A series of characterizations revealed that the α-MoO_3_ belt-like structures had good crystallinity, high purity and good thermal stability as well as luminescence property of 2.578 eV. It may be used in high-temperature devices and has good potential in light-emitting devices, which realized the recycling of discarded molybdenum disilicide heating element.

## Methods

A discarded silicon molybdenum rod was used as the raw material in this experiment, the surface of which was polished to remove the formed oxide before using. Then the silicon molybdenum rod with fresh surface was put on top of a corundum crucible. The set was then placed in a high temperature furnace and heated at 1000 °C in air for 3 h. After furnace-cooling to room temperature, the product deposited in the bottom of the corundum crucible was subjected to detailed characterization using the techniques described below.

The phases of the resultant products were identified by X-ray diffraction (XRD; XD–3, Purkinje General Instrument Co., Ltd) with Cu Kα_1_ radiation (λ = 1.5406 Å). The qualitative information was obtained by X-ray photoelectron spectrometer (XPS, Axis Ultra DLD) and Fourier transformation infrared spectroscopy (FTIR, Thermo Nicolet 5700). Their morphologies and microstructures were observed with both a scanning electron microscope (SEM; JEM–6460 LV microscope, Japan), and a transmission electron microscope (TEM, JEOL JEM-2010, Japan) equipment with an energy dispersive spectroscope (EDS, INCA, Oxford Instrument, UK) and selected area electron diffraction (SAED). The thermogravimetric and differential thermal analysis (TG-DTA, 404F3/200F3) of the products were carried out in air atmosphere on the pan of Al_2_O_3_ at 10 °C min^−1^. The room-temperature photoluminescence (PL) spectra of the as-prepared products were examined by a fluorescence spectrophotometer (Hitachi Model F-7000 FL Spectrophotometer).
